# Protracted venous infusion 5-fluorouracil in combination with subcutaneous interleukin-2 and alpha-interferon in patients with metastatic renal cell cancer: a phase II study

**DOI:** 10.1054/bjoc.2000.1418

**Published:** 2000-10

**Authors:** M J Allen, M Vaughan, A Webb, S Johnston, P Savage, T Eisen, S Bate, J Moore, R Ahern, M E Gore

**Affiliations:** Royal Marsden Hospital, London, UK

**Keywords:** renal cell cancer, interleukin-2, interferon, 5-fluorouracil, biochemotherapy

## Abstract

Our purpose was to assess the activity of alpha-interferon (IFN-α), interleukin-2 (IL-2) and 5 fluorouracil (5FU) administered by protracted venous infusion (PVI) as opposed to bolus injection. 55 patients with advanced renal cell cancer were treated as follows: IL-2 and IFN-α according to the schedule originally described by Atzpodien, with PVI 5FU 200 mg m^–2^ day^–1^ during weeks 5–9. 42 patients (76%) were of moderate or poor prognosis as defined by previous studies. The response rate by intention to treat was 31% (17 of 55, three complete response, 14 partial response; 95% CI = 19–45%) and in evaluable patients (completed one cycle, *n* = 42), it was 40% (95% CI = 26–57%). In addition, 24% (13 of 55) patients achieved disease stabilization. The overall median survival was 11 months with a 1-year survival of 45%. The median survival for evaluable patients was 18 months with 1- and 2-year survivals of 60% and 40% respectively. The median survival of responding patients was 31 months and the three patients achieving complete response remain progression-free at 14+, 18+ and 23+ months. Evaluable patients with poor prognostic features achieved a response rate of 54% and median survival of 18 months. Toxicity was significant yet manageable with 12 patients unable to complete one cycle due to side-effects and 36% experiencing grade 3–4 toxicities. The three on-treatment deaths were considered unlikely to be due to toxicity. The schedule of IFN-α, IL-2 and PVI 5FU has significant activity in advanced renal cell cancer with manageable toxicity. It is of particular interest that this regimen appears to have high activity in fit patients with poor prognostic features. © 2000 Cancer Research Campaign


*British Journal of Cancer* (2000) **83**(8), 980–985
© 2000 Cancer Research Campaign
doi: 10.1054/ bjoc.2000.1418, available online at http://www.idealibrary.com on IDEAL®

### Protracted venous infusion 5-fluorouracil in combination with subcutaneous interleukin-2 and alpha-interferon in patients with metastatic renal cell cancer: a phase II study

**MJ Allen, M Vaughan, A Webb, S Johnston, P Savage, T Eisen, S Bate, J Moore, R Ahern and ME Gore**

Royal Marsden Hospital, London, UK

**Summary** Our purpose was to assess the activity of alpha-interferon (IFN-α), interleukin-2 (IL-2) and 5 fluorouracil (5FU) administered by protracted venous infusion (PVI) as opposed to bolus injection. 55 patients with advanced renal cell cancer were treated as follows: IL-2 and IFN-α according to the schedule originally described by Atzpodien, with PVI 5FU 200 mg $m^{-2}$ $day^{-1}$ during weeks 5–9. 42 patients (76%) were of moderate or poor prognosis as defined by previous studies. The response rate by intention to treat was 31% (17 of 55, three complete response, 14 partial response; 95% CI = 19–45%) and in evaluable patients (completed one cycle, *n* = 42), it was 40% (95% CI = 26–57%). In addition, 24% (13 of 55) patients achieved disease stabilization. The overall median survival was 11 months with a 1-year survival of 45%. The median survival for evaluable patients was 18 months with 1- and 2-year survivals of 60% and 40% respectively. The median survival of responding patients was 31 months and the three patients achieving complete response remain progression-free at 14+, 18+ and 23+ months. Evaluable patients with poor prognostic features achieved a response rate of 54% and median survival of 18 months. Toxicity was significant yet manageable with 12 patients unable to complete one cycle due to side-effects and 36% experiencing grade 3–4 toxicities. The three on-treatment deaths were considered unlikely to be due to toxicity. The schedule of IFN-α, IL-2 and PVI 5FU has significant activity in advanced renal cell cancer with manageable toxicity. It is of particular interest that this regimen appears to have high activity in fit patients with poor prognostic features. © 2000 Cancer Research Campaign

**Keywords**: renal cell cancer; interleukin-2; interferon; 5-fluorouracil; biochemotherapy

Renal cell carcinoma accounts for only 2% of all cancers but its incidence is increasing (Ries et al, 1997). Radical nephrectomy is potentially curative for those with local disease. However, approximately 25% of patients present with metastatic disease and a further 30–40% of patients with disease that is apparently confined to the kidney at presentation will eventually develop systemic spread (Rabinovitch et al, 1994). The prognosis for patients with metastases is very poor and median survival is only 6 months (Selli et al, 1983). Renal cell carcinoma is not chemosensitive, although short-duration responses can be obtained with vinblastine (response rate 7%) and 5-fluorouracil (response rate 10%) (Yagoda et al, 1995). The mainstay of treatment for metastatic renal cell carcinoma is immunotherapy with alpha-interferon (IFN-α) or interleukin-2 (IL-2), both of which have demonstrated response rates of 10–30% (Muss, 1991; Bukowski, 1997). Recently, a randomized MRC trial showed a significant survival advantage for interferon compared to medroxyprogesterone: median survival 8.5 vs 6 months and response rate 14% vs 2% (Medical Research Council Renal Cancer Collaborators, 1999). Some reports have suggested that IL-2 results in slightly higher response rates of 15–30% (Rosenberg et al, 1994; Fyfe et al, 1995) compared with 10–20% for IFN-α (Muss, 1991; Minasian et al, 1993) and longer median survival 16 months (Fyfe et al, 1995) vs 11 months (Minasian et al, 1993)) and that more patients achieve durable complete remissions. However, a randomized study has failed to show any difference in efficacy between these two cytokines (Negrier et al, 1998). In this trial a third arm involved a combination of IFN-α and IL-2 and although higher response rates and longer event-free survival were reported with the combination, there was no overall survival benefit. Thus, current standard therapy for patients with metastatic renal cell carcinoma is either single-agent IFN-α or IL-2 in those fit enough to tolerate the treatment.

The highest response rates in metastatic renal cell cancer have been reported with a combination of IFN-α–IL-2–5FU (bolus) given according to the regimen first described by Atzpodien and colleagues (Atzpodien et al, 1993; 1997; Hofmockel et al, 1996; Ellerhorst et al, 1997). However, the optimal method of delivery of these drugs remains to be established. In a previous study at the Royal Marsden Hospital, we demonstrated the feasibility of combining PVI (protracted venous infusion) 5FU with subcutaneous IL-2 in patients with metastatic renal cell cancer (Savage et al, 1997) and we now report our experience of adding IFN-α 2a to this regimen at the same dose and schedule of IFN-α and IL-2 as that described by Aztpodien et al (1993).

### PATIENTS AND METHODS

### Patients

Patients were eligible for the study if they had histologically proven renal cell carcinoma with metastatic disease. Inclusion

*Received 1 December 1999*
*Revised 13 June 2000*
*Accepted 27 June 2000*

*Correspondence to:* ME Gore criteria were as follows: age > 18 years; Eastern Co-operative Oncology Group (ECOG) performance status (PS) 0–2; bidimensionally measurable disease; normal haematological and biochemical parameters and a glomerular filtration rate greater than 60 ml $min^{-1}$. Patients were excluded if they had any significant other medical illness, previous or concomitant malignancy (excluding cervical carcinoma in situ or basal cell carcinoma of skin), cerebral metastases, or had received chemotherapy, radiotherapy or immunotherapy within the previous 4 weeks. Patients who had received prior biochemotherapy, or who were taking long-term corticosteroids, were excluded. Written informed consent was obtained from all patients in accordance with the Royal Marsden Hospital Research and Ethics Committee guidelines.

Prognostic factors identified in earlier studies (Elson et al, 1988; Palmer et al, 1992; Jones et al, 1993; Fossa et al, 1995) were used to categorize patients into three groups. These factors were: PS > 0; more than one metastatic site; interval from diagnosis of primary tumour to treatment of metastatic disease < 2 years. Patients with 0 or 1 factors were defined as good prognosis, patients with 2 factors as moderate prognosis, and patients with 3 factors as poor prognosis.

### Pre-treatment evaluation

Pre-treatment assessment included full and differential blood count, electrolytes, calcium, liver enzymes, thyroid function tests and coagulation profile. Baseline electrocardiograms and chest radiographs were also performed. Tumour assessment was by computed tomography (CT) scan and/or plain X-rays as appropriate.

### Treatment

A 9-week treatment schedule was employed as in Table 1. Patients were admitted for treatment during weeks 1 and 4. A Hickman line was inserted during week 4 and warfarin was commenced at a dose of 1 mg daily as prophylaxis against Hickman line-associated thrombosis. Patients who received a second or third cycle of treatment continued their PVI 5FU throughout the entire 9 weeks of these cycles.

### Toxicity assessment

Patients were evaluated for toxicity at weeks 1, 4 and 9. Treatment toxicity was assessed using National Cancer Institute of Canada Clinical Trials Group (NCIC-CTG) Expanded Common Toxicity Criteria (CTC). Paracetamol and/or naproxen were used to ameliorate the constitutional side-effects of IFN-α and IL-2. Chlorpheniramine was used to relieve pruritus when it occurred and anti-emetics, sedatives and anti-diarrhoeal agents were given as necessary. Intravenous colloids were used for the initial treatment of IL-2-induced hypotension, followed by dopamine if required. Dose modifications for 5FU toxicity were made as follows: mucositis or palmar-plantar erythema (≥ grade 2) or diarrhoea (≥ grade 3), 5FU stopped until resolution of toxicity and restarted with a 25% dose reduction.

**Table 1** Treatment schedule

| | | | |
|---|---|---|---|
| Weeks 1 and 4 | Day 1 | sc IFN-α 2a | 6 MU $m^{-2}$ |
| | Days 3–5 | sc IL-2 | 10 MU $m^{-2}$ bd |
| Weeks 2 and 3 | Days 1, 3, 5 | sc IFN-α 2a | 6 MU $m^{-2}$ |
| | | sc IL-2 | 5 MU $m^{-2}$ |
| Weeks 5–8 | | PVI 5FU | 200 mg $m^{-2}$ $day^{-1}$ |
| | Days 1, 3, 5 | sc IFN-α 2a | 9 MU $m^{-2}$ |
| Week 9 | Response assessment | | |
| | PVI 5FU continued | | |
| | Option of further cycle in responding patients, commencing week 10 | | |

### Assessment of response

Tumour response was assessed radiologically at week 9 of each cycle. Complete response (CR) was defined as the disappearance of all known disease, partial response (PR) as a 50% or more decrease in the sum of the products of largest and perpendicular diameters of measurable lesions without the appearance of new lesions or progression of any existing lesion, progressive disease (PD) as a 25% or more increase in size of one or more measurable lesions, or the appearance of a new lesion(s) and stable disease (SD) as a < 25% increase or < 50% decrease in the size of measurable lesions without the appearance of new lesions or progression of any existing lesion. Patients who demonstrated CR, PR or SD proceeded to a second cycle of treatment. Patients were subsequently followed up at 3-monthly intervals.

### Statistical analysis

Lifetable curves and median survivals were calculated using the Kaplan–Meier method. Fisher's exact test and the Mann–Whitney test for trend were used to compare proportions.

### RESULTS

### Patient characteristics

The characteristics of the 55 patients who entered the study are shown in Table 2. Forty-seven patients (85%) had more than one disease site at the time of treatment initiation; 26 patients (47%) had an ECOG PS of 1 and two patients (4%) had a PS of 2. The time from diagnosis to treatment of metastatic disease was < 2 years in 41 patients (74.5%). Table 3 shows the distribution of patients according to prognostic group: 13 patients (23.5%) had a good prognosis, 23 patients (42%) had a moderate prognosis and 19 patients (34.5%) were in the poor prognosis group. Thus, 42 patients (76.5%) were in a moderate or poor prognosis group.

### Toxicities

All patients were evaluable for toxicity and Table 4 shows these toxicities according to NCIC-CTG CTC grade (worst toxicity per patient). All patients experienced at least one episode of grade 1 or 2 toxicity. Seventeen patients experienced one or more non-haematological grade 3 toxicity, and grade 4 toxicity was seen in three patients (one patient grade 4 dyspnoea, one patient grade 4 infection and one patient vomiting and hypotension both grade 4).

Patients were electively admitted to hospital during weeks 1 and 4, as this was the time when higher doses of IL-2 were administered. As a result, toxicities during this period were closely monitored. Twelve patients (22%) failed to complete the first course of treatment because of unacceptable toxicity: six of these were in the poor prognosis group, three in the moderate prognosis group and three in the good prognosis group.

© 2000 Cancer Research Campaign

**Table 2** Patient characteristics

| Characteristics | *n* |
|---|---|
| Sex | |
| Male | 44 |
| Female | 11 |
| Age, years | |
| Median | 52 |
| Range | 28–66 |
| Prior nephrectomy | 49 |
| Prior treatment | 7 |
| IL-12 | 3 |
| IFN | 3 |
| Radiotherapy | 1 |
| ECOG performance status | |
| 0 | 27 |
| 1 | 26 |
| 2 | 2 |
| No. of disease sites | |
| 1 | 8 |
| 2 | 27 |
| 3 | 12 |
| 4 | 6 |
| 5 | 2 |
| Time from diagnosis to treatment of metastatic disease | |
| 0–12 mths | 30 |
| 12–24 mths | 11 |
| >24 mths | 14 |
| Sites of metastases | |
| Lung | 39 |
| Bone | 12 |
| Lymph nodes | 29 |
| Liver | 7 |
| Renal bed | 21 |

**Table 3** Prognostic groups

| Prognostic group | Poor prognostic factors[a] (*n*) | Patients (*n*) |
|---|---|---|
| Good | 0 or 1 | 13 |
| Moderate | 2 | 23 |
| Poor | 3 | 19 |

[a]Prognostic factors = PS > 0; > 1 site of disease; < 2 years from primary diagnosis to treatment of metastatic disease

**Table 4** Toxicity

| | CTC Grade (worst per patient) | | | | |
|---|---|---|---|---|---|
| | 0 | 1 | 2 | 3 | 4 |
| Cytokine-related | | | | | |
| Malaise | 6 | 20 | 24 | 5 | 0 |
| Rigors | 17 | 31 | 7 | 0 | 0 |
| Skin flushing | 9 | 29 | 15 | 2 | 0 |
| Hypotension | 30 | 6 | 15 | 3 | 1 |
| Oedema | 36 | 13 | 6 | 0 | 0 |
| Dyspnoea | 43 | 4 | 4 | 3 | 1 |
| Vomiting | 26 | 12 | 14 | 2 | 1 |
| Nausea | 17 | 14 | 18 | 6 | 0 |
| Myalgia | 23 | 24 | 8 | 0 | 0 |
| 5-FU-related | | | | | |
| Stomatitis | 40 | 6 | 7 | 2 | 0 |
| Diarrhoea | 32 | 11 | 12 | 0 | 0 |
| Haematological | | | | | |
| Anaemia | 19 | 1 | 33 | 2 | 0 |
| Leucopenia | 36 | 12 | 7 | 0 | 0 |
| Thrombocytopenia | 51 | 0 | 1 | 3 | 0 |
| Neutropenia | 32 | 6 | 10 | 7 | 0 |
| Infection | 46 | 5 | 2 | 1 | 1 |

Three patients died while on treatment. One patient died of a haemorrhagic cerebrovascular accident during week 9 of her second cycle of treatment. She had been anticoagulated with warfarin following a diagnosis of pulmonary embolism 4 months prior to starting treatment. The second patient died during his third cycle of treatment from previously undiagnosed progressive cerebral metastases associated with oedema which caused tentorial herniation. Post-mortem examination was not undertaken in accordance with the wishes of the next-of-kin. The third patient was admitted to her local hospital with a history of sudden-onset dyspnoea during week 9 of the first cycle of treatment, before any response assessment had been carried out. She died on the day of admission and although no post-mortem was performed, the clinical diagnosis was pulmonary embolism.

### Response to treatment and survival

Response to treatment was analysed by intention to treat and the overall response rate was 31% (17 of 55; 95% CI = 19–45%) with a complete response rate of 5.4% (3 of 55; 95% CI = 1.1–15%) and a partial response rate of 25% (14 of 55; 95% CI = 15–39%). Stabilization of disease was seen in a further 13 patients (23.6%). The response rate was 15% in the good prognosis group, 35% in the moderate prognosis group, and 37% in the poor prognosis group. Overall median progression-free survival was 20 weeks (range 1–98+) and median duration of response was 73 weeks (range 6–88+). The median survival by intention to treat was 47 weeks (range 2–134) with no significant differences between prognostic groups. The 1-year survival was 45% overall; interestingly 33% for the good prognosis group and 45% for poor prognosis patients.

An analysis of evaluable patients (Table 5) was also undertaken. This excluded patients in whom response could not be assessed, i.e. those who failed to complete one cycle of treatment because of unacceptable toxicity and the patient who died during the first cycle. There were 42 evaluable patients and the response rate in this group was 40% (17 of 42; 95% CI = 26–57%). Their median survival was 79 weeks (range 12–134), 1-year survival 60% and 2-year survival 40%. The median survival of responding patients was 30.9 months (range 4.1–30.9), and the three patients who achieved CR remain progression-free at their last follow up at 14+, 18+ and 23+ months respectively. Their clinical features are shown in Table 6.

We did not find that response was associated with good prognosis group (Table 5). On the contrary, there was a trend towards a higher response rate and longer survival in the poor prognosis group, although this did not reach statistical significance.

### DISCUSSION

The optimal treatment regimen for patients with metastatic renal cell cancer remains to be established. The highest response rates have been reported for a combination of IFN-α–IL-2–5FU (bolus). The combination of subcutaneous IFN-α–IL-2 with PVI 5FU as reported here utilizes the same doses and schedule of IFN-α and IL-2 as that described by Atzpodien and colleagues for the

 *© 2000 Cancer Research Campaign*

**Table 5** Response and survival in evaluable patients

| | Prognostic group | | | |
|---|---|---|---|---|
| | **Good (*n* = 10)** | **Moderate (*n* = 19)** | **Poor (*n* = 13)** | **Overall (*n* = 42)** |
| Response rate | 20% | 42% | 54% | 40% |
| Median duration of response (weeks) | 6 | 21 | NR | 73 |
| Median survival (weeks) | 51 | 87 | 79 | 79 |
| 1-year survival | 51% | 62% | 64% | 60% |

NR = not reached

IFN-α–IL-2–5FU (bolus) combination. We have shown that IFN-α–IL-2–PVI 5FU is feasible and results in high response rates that are comparable with those seen in some other studies of this combination (Atzpodien et al, 1993; 1997; Hofmockel et al, 1996; Ellerhorst et al, 1997). These encouraging results are of particular interest because of the relatively poor prognostic features of our patients.

The reported response rates to IFN-α–IL-2–5FU (bolus) vary widely from 1.8–48.6% (Atzpodien et al, 1993; 1997; Dutcher et al, 1996; Hofmockel et al, 1996; Ellerhorst et al, 1997; Negrier et al, 1997; Ravaud et al, 1998; Tourani et al, 1998) (Table 7) and two main factors probably account for this. First, the patient characteristics of the study populations and secondly, differences in the scheduling of the drugs. For instance, Ravaud and colleagues reported that a combination of interferon, IL-2 and 5FU was inactive with a response rate of only 1.8% (Ravaud et al, 1998). The scheduling and dosing of the agents in this study was very different to the regimen as originally described by Atzpodien and colleagues with IFN-α and IL-2 total doses of 72 MU $m^{-2}$ and 216 MU $m^{-2}$ respectively, as opposed to 156 MU $m^{-2}$ and 150 MU $m^{-2}$. Furthermore, in the Ravaud study IL-2 was given subcutaneously for 6 days every other week for 8 weeks, whereas it was given on days 3, 4 and 5 of weeks 1 and 4 and days 1, 3 and 5 of weeks 2 and 3 in the Aztpodien regimen. IFN-α is administered on day 1 of weeks 1 and 4, and days 1, 3 and 5 of weeks 2 and 3 and 5–8 of the Aztpodien regimen, but was given on days 1, 3 and 5 with IL-2 for 8 weeks in the Ravaud trial. Aztpodien gave bolus 5-FU once weekly in weeks 5–8, while Ravaud gave an infusion of 5-FU for 5 days every 4 weeks, starting in week 1. On the other hand, Ellerhorst et al (1997) and Tourani et al (1998) use drug schedules which are quite different again but achieve better response rates than Ravaud. Thus, the scheduling of these drugs (including doses, sequencing, drug combination and modes of administration) is quite different between regimens and may account in some part for the reported differences in activity.

**Table 6** Clinical features of complete responders

| | Disease sites | Prognosis group | Response duration |
|---|---|---|---|
| Patient 20 | Lung<br>Lymph nodes | Poor | 80+ weeks |
| Patient 21 | Lung | Poor | 98+ weeks |
| Patient 45 | Lung<br>Lymph nodes | Poor | 59+ weeks |

The importance of patient characteristics is demonstrated by several studies that have identified a number of factors which have an impact on the survival of patients with metastatic renal cell cancer following treatment with IFN-α, IL-2 and chemotherapy (Elson et al, 1988; Palmer et al, 1992; Jones et al, 1993; Fossa et al, 1995). These prognostic factors are ECOG performance status, time from diagnosis to treatment and the number of metastatic sites (Table 3). Joffe et al (1996) reported lower response rates (17%) to IFN-α–IL-2–5FU (bolus) than Atzpodien and colleagues (48.6%) but the former study included a high proportion of moderate (27%) and poor (56%) prognosis patients. Another example is the recent randomized trial comparing IFN-α–IL-2–5FU (bolus) with single-agent tamoxifen (Atzpodien et al, 1997), which reported that the median survival of the patients treated in the tamoxifen arm was 14 months. This is considerably better than the median survival reported for IFN-α-treated patients in the randomized MRC trial of IFN-α vs medroxyprogesterone (8.5 months vs 6 months (Medical Research Council Renal Cancer Collaborators, 1999)). These data suggest that the clinical characteristics of patients in a study may have a profound effect on the results. The high response rate reported here is of particular interest because we have demonstrated that our regimen is active in poor-prognosis patients and three of these patients have obtained durable CRs. Furthermore, the median survival of poor-prognosis patients in our series is similar to those with good prognostic features. We have described above how Atzpodien and colleagues mainly treated good-prognosis patients and demonstrated high response rates, while Joffe failed to confirm this activity in a population of poorer-prognosis patients. Our results confirm those of Atzpodien but in a similar patient population to those treated by Joffe and colleagues. A possible explanation for this is our use of PVI 5FU, as opposed to its bolus administration.

**Table 7** IFN-α–IL-2–5FU studies

| First author, year | *n* | Response-rate | Median survival | Prognostic features | Atzpodien regimen |
|---|---|---|---|---|---|
| Allen, 2000 | 55 | 31% | 10.7 mths | Poor | Yes |
| Atzpodien, 1993 | 35 | 49% | N/A | Good | Yes |
| Atzpodien, 1997 | 41 | 39% | 42 mths | Good | Yes |
| Hofmockel, 1996 | 34 | 38% | N/A | N/A | Yes |
| Ellerhorst, 1997 | 55 | 31% | 23 mths | Moderate | No |
| Tourani, 1998 | 62 | 19% | 33% at 2 yrs | Moderate | No |
| Dutcher, 1996 | 36 | 19% | N/A | Moderate | Yes |
| Joffe, 1996 | 55 | 17% | 12 mths | Poor | Yes |
| Negrier, 1997 | 61 | 8% | N/A | N/A | No |
| Ravaud, 1998 | 111 | 2% | 12 mths | Poor | No |

[a]Randomized; mths = months; N/A = not available

© 2000 Cancer Research Campaign

5FU, an anti-metabolite, is active principally in the S-phase of the cell cycle and therefore may be more effective when given as a protracted venous infusion rather than as a bolus injection, by increasing the proportion of tumour cells in S-phase which are exposed to 5FU. This may be particularly important for tumours with a relatively slow doubling time. In addition, PVI 5FU enables higher dose-intensity of the drug than intravenous bolus administration. PVI 5FU-containing regimens have been associated with high response rates in breast cancer (Smith et al, 1995) and relapsed ovarian cancer (Ahmed et al, 1996), improved response rates and survival in colorectal cancer (Lokich et al, 1989; Meta-analysis Group in Cancer, 1998) and a survival advantage over a standard 5FU-containing regimen in oesophagogastric cancer (Webb et al, 1997).

The IFN-α–IL-2–PVI 5FU regimen that we describe here is associated with significant toxicity with 36% of patients experiencing grade 3 or 4 toxicity, but most episodes were manageable with appropriate supportive measures. Interestingly, in our study 12 patients (22%) discontinued treatment before completing their first cycle because of unacceptable toxicity. Half of these patients were among the first 15 treated, suggesting that with experience we became better at managing the toxicities associated with this regimen and identifying those individuals who would be more likely to tolerate treatment.

From our results and a review of the other phase II studies combining IFN-α–IL-2–5FU, it is clear that, although there is an element of empiricism in the cytokine schedule, when this is altered response rates appear to fall dramatically (Table 7). The regimen can be associated with significant toxicity in many patients but the side-effects are manageable in the majority. We would make the following recommendations concerning further studies involving IFN-α–IL-2–5FU: randomization against standard therapy (IFN-α or IL-2 as single agents); inclusion of fit patients with poor prognostic features; the administration of 5FU in a prolonged schedule (PVI 5FU or one of the new oral preparations) should be further explored. In the future, maintenance cytokine schedules need to be developed.

### REFERENCES

Ahmed FY, Mainwaring PN, MacFarlane V, King DM, Mansi J, Slevin M, Gallagher C, Harper P and Gore ME (1996) Infusional chemotherapy (cisplatin, epirubicin, 5FU, ECF) for patients with epithelial ovarian carcinoma. 9th NCI-EORTC Symposium on New Drug Therapy, Amsterdam.

Atzpodien J, Kirchner H, Hanninen EL, Deckert M, Fenner M and Poliwoda H (1993) Interleukin-2 in combination with interferon-alpha and 5-fluorouracil for metastatic renal cell cancer. *Eur J Cancer* **29A** (suppl. 5): S6–8

Atzpodien J, Kirchner H, Franzke A, Wandert T, Probst M, Buer J, Duensing S and Ganser A (1997) Results of a randomized clinical trial comparing SC interleukin-2, SC alpha-2a-interferon, and IV bolus 5-fluorouracil against oral tamoxifen in progressive metastatic renal cell carcinoma patients (Meeting abstract). *Proc Ann Meet Am Soc Clin Oncol* **16**: 00–00

Bukowski RM (1997) Natural history and therapy of metastatic renal cell carcinoma: the role of interleukin-2. *Cancer* **80**: 1198–1220

Dutcher J, Logan T, Gordon M, Sosman J, Weiss G, Margolin K, Plasse T and Atkins M (1996) 5FU + subcutaneous (sc) interleukin-2 (IL2) plus sc intron (IFN) in metastatic renal cell cancer (RCC) patients (pts). A CWG study. *Proc Ann Meet Am Soc Clin Oncol* **15**: A725

Ellerhorst JA, Sella A, Amato RJ, Tu SM, Millikan RE, Finn LD, Banks M and Logothetis CJ (1997) Phase II trial of 5-fluorouracil, interferon-alpha and continuous infusion interleukin-2 for patients with metastatic renal cell carcinoma. *Cancer* **80**: 2128–2132

Elson PJ, Witte RS and Trump DL (1988) Prognostic factors for survival in patients with recurrent or metastatic renal cell carcinoma. *Cancer Res* **48**: 7310–7313

Fossa S, Jones M, Johnson P, Joffe J, Holdener E, Elson P, Ritchie A and Selby P (1995) Interferon-alpha and survival in renal cell cancer. *Br J Urol* **76**: 286–290

Fyfe G, Fisher RI, Rosenberg SA, Sznol M, Parkinson DR and Louie AC (1995) Results of treatment of 255 patients with metastatic renal cell carcinoma who received high-dose recombinant interleukin-2 therapy. *J Clin Oncol* **13**: 688–696

Hofmockel G, Langer W, Theiss M, Gruss A and Frohmuller HG (1996) Immunochemotherapy for metastatic renal cell carcinoma using a regimen of interleukin-2, interferon-alpha and 5-fluorouracil. *J Urol* **156**: 18–21

Joffe JK, Banks RE, Forbes MA, Hallam S, Jenkins A, Patel PM, Hall GD, Velikova G, Adams J, Crossley A, Johnson PW, Whicher JT and Selby PJ (1996) A phase II study of interferon-alpha, interleukin-2 and 5-fluorouracil in advanced renal carcinoma: clinical data and laboratory evidence of protease activation. *Br J Urol* **77**: 638–649

Jones M, Philip T, Palmer P, von der Maase H, Vinke J, Elson P, Franks CR and Selby P (1993) The impact of interleukin-2 on survival in renal cancer: a multivariate analysis. *Cancer Biother* **8**: 275–288

Lokich JJ, Ahlgren JD, Gullo JJ, Philips JA and Fryer JG (1989) A prospective randomized comparison of continuous infusion fluorouracil with a conventional bolus schedule in metastatic colorectal carcinoma: a Mid-Atlantic Oncology Program Study. *J Clin Oncol* **7**: 425–432

Medical Research Council Renal Cancer Collaborators (1999) Interferon-alpha and survival in metastatic renal carcinoma: early results of a randomised controlled trial. *Lancet* **353**: 14–17

Meta-analysis Group in Cancer (1998) Efficacy of intravenous continuous infusion of fluorouracil compared with bolus administration in advanced colorectal cancer. *J Clin Oncol* **16**: 301–308

Minasian LM, Motzer RJ, Gluck L, Mazumdar M, Vlamis V and Krown SE (1993) Interferon alfa-2a in advanced renal cell carcinoma: treatment results and survival in 159 patients with long-term follow-up. *J Clin Oncol* **11**: 1368–1375

Muss HB (1991) The use of interferon in renal cell carcinoma. *Eur J Cancer* **27**: S84–S87

Negrier S, Escudier B, Douillard JY, Lesimple T, Rossi JF, Viens P, DiStefano-Louineau D, Drevon M, Gomez F and Caty A (1997) Randomized study of interleukin-2 (IL2) and interferon (IFN) with or without 5-FU (FUCY study) in metastatic renal cell carcinoma (MRCC). *Proc Ann Meet Am Soc Clin Oncol* **16**: A1161

Negrier S, Escudier B, Lasset C, Douillard JY, Savary J, Chevreau C, Ravaud A, Mercatello A, Peny J, Mousseau M, Philip T and Tursz T (1998) Recombinant human interleukin-2, recombinant human interferon alfa-2a, or both in metastatic renal-cell carcinoma. Groupe Francais d'Immunotherapie. *N Engl J Med* **338**: 1272–1278

Palmer PA, Vinke J, Philip T, Negrier S, Atzpodien J, Kirchner H, Oskam R and Franks CR (1992) Prognostic factors for survival in patients with advanced renal cell carcinoma treated with recombinant interleukin-2. *Ann Oncol* **3**: 475–480

Rabinovitch RA, Zelefsky MJ, Gaynor JJ and Fuks Z (1994) Patterns of failure following surgical resection of renal cell carcinoma: implications for adjuvant local and systemic therapy. *J Clin Oncol* **12**: 206–212

Ravaud A, Audhuy B, Gomez F, Escudier B, Lesimple T, Chevreau C, Douillard JY, Caty A, Geoffrois L, Ferrero JM, Linassier C, Drevon M and Negrier S (1998) Subcutaneous interleukin-2, interferon alfa-2a, and continuous infusion of fluorouracil in metastatic renal cell carcinoma: a multicenter phase II trial. Groupe Francais d'Immunotherapie. *J Clin Oncol* **16**: 2728–2732

Ries LAG, Kosary CL, Hankey BF, Miller BA, Harras A and Edwards BK (eds) (1997) *SEER cancer statistics review, 1973–1994* National Cancer Institute: Bethesda (NIH Publication 97–2789)

Rosenberg SA, Yang JC, Topalian SL, Schwartzentruber DJ, Weber JS, Parkinson DR, Seipp CA, Einhorn JH and White DE (1994) Treatment of 283 consecutive patients with metastatic melanoma or renal cell cancer using high-dose bolus interleukin 2. *JAMA* **271**: 907–913

Savage P, Costelna D, Moore J and Gore ME (1997) A phase II study of continuous infusional 5-fluorouracil (5-FU) and subcutaneous interleukin-2 (IL-2) in metastatic renal cancer. *Eur J Cancer* **33**: 1149–1151

Selli C, Hinshaw WM, Woodard BH and Paulson DF (1983) Stratification of risk factors in renal cell carcinoma. *Cancer* **52**: 899–903

Smith IE, Walsh G, Jones A, Prendiville J, Johnston S, Gusterson B, Ramage F, Robertshaw H, Sacks N, Ebbs S, McKinna JA and Baum M (1995) High complete remission rates with primary neoadjuvant infusional chemotherapy for large early breast cancer. *J Clin Oncol* **13**: 424–429

Tourani JM, Pfister C, Berdah JF, Benhammouda A, Salze P, Monnier A, Paule B, Guillet P, Chretien Y, Brewer Y, Di Palma M, Untereiner M, Malaurie E, Tadrist Z, Pavlovitch JM, Hauteville D, Mejean A, Azagury M, Mayeur D, Lucas V, Krakowski I, Larregain-Fournier D, Abourachid H, Andrieu JM and

 © 2000 Cancer Research Campaign

Chastang C (1998) Outpatient treatment with subcutaneous interleukin-2 and interferon alfa administration in combination with fluorouracil in patients with metastatic renal cell carcinoma: results of a sequential nonrandomized phase II study. Subcutaneous Administration Propeukin Program Cooperative Group. *J Clin Oncol* **16**: 2505–2513

Webb A, Cunningham D, Scarffe JH, Harper P, Norman A, Joffe JK, Hughes M, Mansi J, Findlay M, Hill A, Oates J, Nicolson M, Hickish T, O'Brien M, Iveson T, Watson M, Underhill C, Wardley A and Meehan M (1997) Randomized trial comparing epirubicin, cisplatin, and fluorouracil versus fluorouracil, doxorubicin, and methotrexate in advanced esophagogastric cancer. *J Clin Oncol* **15**: 261–267

Yagoda A, Abi-Rached B and Petrylak D (1995) Chemotherapy for advanced renal-cell carcinoma: 1983–1993. *Semin Oncol* **22**: 42–60

© 2000 Cancer Research Campaign
